# Type-1 inflammatory imprinting and programmed responsiveness to CD40L enhance Siglec-1-dependent HIV-1 trans-infection by dendritic cells

**DOI:** 10.3389/fimmu.2026.1834769

**Published:** 2026-06-04

**Authors:** E. Grace Bothwell, Allison E. DePuyt, Colleen R. Zaccard, Renee. R. Anderko, Peter E. J. Shoucair, Holly A. Bilben, Tatiana M. Garcia-Bates, Abigail D. Gerberick, Simon C. Watkins, Nicolas Sluis-Cremer, Charles R. Rinaldo, Robbie B. Mailliard

**Affiliations:** 1 Department of Infectious Diseases and Microbiology, University of Pittsburgh School of Public Health, Pittsburgh, PA, United States; 2 Department of Surgery, University of Pittsburgh School of Medicine, Pittsburgh, PA, United States; 3 Department of Medicine, University of Pittsburgh School of Medicine, Pittsburgh, PA, United States; 4 Department of Cell Biology, University of Pittsburgh School of Medicine, Pittsburgh, PA, United States

**Keywords:** CD40L, dendric cells (DCs), HIV-1 trans-infection, Siglec 1, tunneling nanotubes (TNT)

## Abstract

Dendritic cells (DC) play a central role in host immunity as they carry environmental cues from sites of infection to the lymphatics, where they subsequently direct appropriate adaptive immune responses. Importantly, this process is exploited by pathogens such as HIV-1, which utilize DC to efficiently facilitate HIV-1 *trans*-infection of CD4^+^ T cells. In this study, we show that monocyte-derived DC matured under type-1 proinflammatory conditions, either through exposure to soluble mediators of type-1 immunity or through bystander activation of cytotoxic T cells, display enhanced *trans*-infection capacity, while prostaglandin E2 exposure diminishes this trait. This heightened *trans*-infection activity involves DC upregulation of sialic acid binding immunoglobulin-like lectin-1 (Siglec-1/CD169) surface expression and a distinct responsiveness to the CD4^+^ T helper cell signal CD40L. The upregulated expression of Siglec-1 increases HIV-1 surface binding potential on these proinflammatory DC, while CD40L/CD40 signaling uniquely induces the formation of pronounced morphologic extensions and the release of the chemokine CCL20, together increasing CD4+ T cell access and susceptibility to HIV-1 infection. Overall, this study demonstrates that the nature of environmental signals received by monocyte-derived DC during maturation and the character of their subsequent responsiveness to CD40L-expressing T helper cells dictates their ability to facilitate HIV-1 *trans*-infection.

## Introduction

As professional antigen-presenting cells, dendritic cells (DC) bridge the gap between the innate and adaptive branches of immunity. They carry pathogen and tissue derived information from the peripheral environment to lymphoid tissues where this is subsequently communicated to induce an appropriate adaptive immune response carried out by the effector functions of T cells and B cells. This process of environmental immune instruction involves immature DC sensing pathogen-associated molecular patterns (PAMPs) as well as cytokines released by neighboring cells, which promptly transforms their phenotypic and functional status as mature DC (1–3). It is well established that the capacity for DC to effectively communicate and induce a necessary adaptive immune response is heavily influenced by the nature of the signals they receive during maturation. Additionally important are the outcomes of DC cognate interaction with CD40L-expressing CD4^+^ T cells, where ligation of CD40 on DC enhances their survival, their antigen presenting function, and subsequently triggers their release of cytokines and chemokines (4, 5). The contribution of CD4^+^ T cell help in unleashing the programmed function of mature DC marks CD40L as a critical signal for boosting their therapeutic potential (6).

Besides functioning as central antigen presenting cells of the immune system, DC can also be utilized or manipulated by pathogens for their survival advantage. An example of this is in the setting of HIV-1 infection, where DC contribute to HIV-1 dissemination through a process termed *trans*-infection. During *trans-*infection, DC facilitate HIV-1 transfer and subsequent infection of CD4^+^ T cells (7). DC-mediated HIV-1 *trans*-infection is considerably more efficient than the direct, cell-free transmission of virus (*cis*-infection) to CD4^+^ T cells, and thus many studies have investigated both the mechanisms of *trans*-infection by DC as well as its contribution to viral pathology *in vivo* (8–14). During the *trans*-infection process, DC first capture infectious HIV-1 using surface proteins that enable trafficking of the virus within or along the surface of DC and, subsequently, deliver the virus to CD4^+^ T cells. The sialic acid-binding Ig-like lectin 1 (Siglec-1/CD169) has been consistently implicated in mediating virus capture by mature DC, although other surface proteins are known to contribute to HIV-1 capture (15–18). Similarly, there are several dissemination mechanisms currently thought to play a role in *trans*-infection, including release of a virus-containing compartment (VCC) at the synapse between a DC and CD4^+^ T cell, virus-containing exosome release, and transfer of HIV-1 bound to tunneling-nanotubes (TNTs) (11, 19–23). However, little information exists on how the nature of the environmental cues received by DC during maturation, as well as the character of their subsequent responsiveness to CD4^+^ T helper cells, might influence the ability of DC to mediate HIV-1 *trans*-infection.

We have previously shown that the establishment of HIV-1 epitope variants can act as partial agonists to existing/cross-reactive CTL, resulting in the selective induction of their release of inflammatory cytokines in the absence of effective target killing (24, 25). This CTL helper activity in turn creates an immune environment promoting the generation of mature pro-inflammatory monocyte-derived DC that are highly efficient at facilitating HIV-1 *trans*-infection of CD4^+^ T cells. These CTL-matured monocyte-derived DC (MDC_CTL_) display traits similar to previously described monocyte-derived DC matured by exposure to mediators of type-1 immunity (MDC1) (26, 27), which includes their high IL-12p70 production and their unique capacity to form TNT networks in response to the T-helper signal CD40L (23). Considering that MDC_CTL_ display an enhanced capacity for *trans*-infection and that they share similar traits to MDC1, which include their distinct responsiveness to the Th signal CD40L, we sought to determine whether the noted enhanced HIV-1 *trans-*infection capacity is a general trait of DC matured under proinflammatory type-1 polarizing conditions, and if CD40/CD40L interaction plays a role this process.

In the current study, we demonstrate how environmental immune instruction dictates the ability of type-1 MDC to efficiently *trans*-infect HIV-1 to CD4^+^ T cells. We show that exposure of immature monocyte-derived DC (iMDC) to pro-inflammatory signals derived from activated CTL or in the form of a cytokine-based activation cocktail designed to mimic the environment of an acute viral infection each drive a type-1 MDC phenotype with high Siglec-1 expression that confers their ability to capture HIV-1 and facilitate its transfer. Additionally, we implicate the mechanistic involvement of CD40L in the *trans-*infection process, wherein proinflammatory type-1 MDC responding to the Th signal form TNT networks that, when blocked, result in a loss of *trans-*infection ability. We also reveal that this type-1 immune instruction uniquely imprints the MDC with the ability to release the chemokine CCL20 following CD40L signaling and confirm that this DC-associated chemokine enhances the susceptibility of the CD4^+^ T cells to HIV-1 infection.

## Materials and methods

### Isolation of human primary cells

PBMC derived from buffy coat blood products purchased from Vitalant were isolated through standard density gradient separation using Lymphocyte Separation Medium (Corning). Primary monocytes were isolated from PBMC with a positive selection human CD14 microbead kit (Miltenyi Biotec) using the manufacturer’s protocol. Following isolation of the monocytes, these along with the remaining peripheral blood lymphocytes were cryopreserved separately until use. Differentially isolated cells were cryopreserved until use. Primary CD8^+^ and CD4^+^ T lymphocytes were separated from cryopreserved peripheral blood lymphocytes using negative magnetic bead separation using EasySep Human CD8^+^ T cell negative selection kit or Human CD4^+^ T cell negative selection kit (STEMCELL Technologies).

### Generation and activation of monocyte-derived DC

Immature monocyte-derived DC (iMDC) were generated from monocytes cultured for 5 days at 37 °C using granulocyte-monocyte colony stimulating factor (GM-CSF, 1000IU/mL, Sanofi-aventis) and interleukin (IL)-4 (1000IU/mL, R&D Systems) in Iscove’s Modified Dulbecco’s Media (IMDM, Gibco) supplemented with 10% fetal bovine serum (FBS, Atlanta Biologicals) and 0.5% gentamicin (Gibco) (complete (c)-IMDM). On day 5, iMDC were differentially exposed to activation factors for 48h before harvest of MDC types for additional experiments on day 7. Mature high IL-12p70 producing MDC1 were generated by adding a previously described cytokine cocktail consisting of IFNα (1000IU/mL, Schering Corporation), IFNγ (1000IU/mL, R&D Systems), IL-1β (10ng/mL, R&D Systems), tumor necrosis factor-α (25ng/mL, R&D Systems), and polyinosinic:polycytidylic acid (poly(I:C)), 20ng/mL, Sigma-Aldrich) (26, 27). Mature IL-12p70 deficient MDC (MDC_PGE2_) were generated by adding another previously described cytokine cocktail consisting of IL-1β (10ng/mL), TNFα (25ng/mL), IL-6 (1000IU/mL, R&D Systems), and prostaglandin-E2 (PGE2, 2 uM Sigma-Aldrich) (28). MDC_CTL_ were generated by adding CD8^+^ T cells at a 1:1 ratio. To mimic antigen specific T cell crosstalk with DC, the T cell-activating human CD3 mAb (OKT3, 1µg/mL, Biolegend) was added to the iMDC just prior addition of the T cells to the DC: CD8^+^ T cell cocultures. Doing so promotes OKT3 binding to the iMDC via FC-receptor and subsequent TCR (anti-CD3) specific engagement and signaling of the CD8^+^ T cells as previously described (29).

### Flow cytometry

Surface protein expression on DC was determined by flow cytometry using cells stained with antibodies for CD83 (PE, clone HB15A, Beckman Coulter), CD86 (PE, clone HA5.2B7, Beckman Coulter), CCR7 (FITC, clone 150503, R&D Systems), OX40L (PE, clone IK-1, BD Biosciences), CD40 (PE, clone MAB89, Beckman Coulter), Siglec-1 (PE, clone 7-239, BioLegend), ICAM-1 (CD54 FITC, clone 84H10, Beckman Coulter), DC-SIGN (CD209-APC, clone DCN46; BD Biosciences). Surface stains were added while cells were in FACS buffer containing 1X PBS (GE Life Sciences) and 0.5% BSA (Sigma-Aldrich). For intracellular HIV p24 detection, cells were surface stained with Aqua Live/Dead^®^ Fixable Dead Cell Stain (Molecular Probes, Life Technologies) followed by surface staining for CD3 (APC-H7, clone SK7, BD Biosciences), CD4 (Pacific Blue, clone RPA-T4, BD Biosciences), and CD8 (PerCP-Cy5.5, clone SK1, BD Biosciences). The cells were then permeabilized with 2X FACS™ Permeabilizing Solution 2 (BD Biosciences) followed by intracellular staining with KC-57, an antibody with broad specificity for the 55, 39, 23, and 24 kDA proteins of the HIV-1 core antigen (FITC, clone FH190-1-1, Beckman Coulter) prior to fixation with 2% PFA and flow cytometric analysis. In all cases, pre-activated, uninfected T cells alone, as well as those co-cultured with relevant DC types in the absence of HIV-1 were stained with the HIV-1 p24 core Ag antibody, which served as negative controls. The cells were then analyzed by flow cytometry. Mock results were subtracted from the experimental group results to yield the recorded values. Analysis for flow cytometry experiments was conducted with the LSRFortessa flow cytometer (BD Biosciences) and FlowJo version 10.10 program, with gating strategy shown in **Supplementary Figure S1**.

### HIV-1 binding assays

Capture of HIV-1 by MDC was determined using a replication deficient HIV-1 expressing GFP on the virion surface (HIV-GFP). HIV-GFP was generated with a plasmid obtained through the NIH AIDS Reagent Program, Division of AIDS, NIAID, NIH: HIV Gag-iGFP_JFRL from Dr. Benjamin Chen (Cat# 12456). The virus was prepared as previously described (30), and titer was quantified on TZM-bl cells. MDC were incubated with HIV-GFP at an MOI of 10–^1^ for 2h at 37˚C followed by extensive washing to remove unbound virus. To block Siglec-1, MDC1 were exposed to anti-Siglec-1 blocking antibody (HSn-7D2, 10µg/mL, Invitrogen) or IgG isotype control (MOPC-21,10µg/mL, Sigma-Aldrich) for 45 minutes at RT prior to incubation with virus. Capture was measured by flow cytometry.

### HIV-1 *cis-* and *trans-*infections

CD4^+^ T cells were cultured at a density of 1x10^6^/mL in RPMI 1640 medium containing 10% FBS, 5% GlutaMax (Gibco), 2.5% gentamycin and activated for 48h using IL-2 (Proleukin^®^, 1000IU/mL, Prometheus Laboratories, Inc.) and phytohemagglutinin (PHA) (1μg/mL, Sigma-Alridch). An R5-tropic strain of HIV-1, HIV-1 BaL, was used to infect CD4^+^ T cells. Briefly, HIV-1 BaL was propagated in and purified from PM1 cells as described (31). For *trans*-infection, MDC were exposed to an MOI of 10–^3^ for 2h at 37 °C followed by extensive washing to remove unbound virus. The MDC (1x10^4^) were co-cultured with autologous CD4^+^ T cells (1x10^5^) in a 96-well round bottom plate in duplicate at a final volume of 200µL. On day 6 of coculture, CD4^+^ T cells were harvested and stained for intracellular p24 protein for flow cytometry. For *cis*-infection, CD4^+^ T cells were pre-exposed for 48h to soluble CCL20 (0.2ng/mL, R&D Systems) or 100µL of CD40L-activated MDC1 supernatants sourced from one culture. For CCL20 blocking experiments, supernatant was incubated for 45 minutes with an anti-CCL20 blocking antibody (10μg/mL, R&D Systems) or IgG isotype control (10μg/mL, R&D Systems). After 48h, CD4^+^ T cells were harvested and infected with HIV-1 at an MOI of 10–^1^ for 2h at 37 °C. Following infection, CD4^+^ T cells were washed extensively to remove unbound virus and placed back into culture with fresh media. On day 2 of culture, cells were treated with IL-2 (1000U/mL) and DynaBeads^®^ Human T-activator CD3/CD28 (Life Technologies) according to manufacturer’s protocol. On day 6, cells were harvested and stained for intracellular p24 protein for flow cytometry.

### Standard and high-resolution microscopy

Standard bright field images were collected using a Leica DM IL LED using a Leica HI Plan I Ph2 40x objective lens (NA = 0.5) using a Leica EC3 Camera system, and images were analyzed with Leica Application Suite software. For imaging MDC1:CD4^+^ T cell co-cultures, MDC1 (1 ×10^5^ cells/ml) and with CD4^+^ T cells (3 ×10^5^ cells/ml) were placed in co-cultures in the presence or absence of staphylococcus enterotoxin B (1 ug/ml) or OKT3 antibody (1ug/ml) as previously described (29). When used, CD40L-specific blocking mAb (Enzo Life Sciences) or control mAb of the same isotype (BD Biosciences, San Jose, CA).

For scanning electron microscopy (SEM), sterile 12-mm round glass coverslips were added to wells in a 48-well plate followed by the addition of MDC1 in base cell culture medium cIMDM at a concentration of 1x105 cells/200µL. Cells were placed in incubator at 37 °C for 1h then additional media containing rhCD40L (0.5µg/mL, MEGACD40L® Protein, Enzo Life Sciences) was added and placed back into incubator for 24h. Cells were prepared for SEM as previously described (24). Briefly, coverslips were removed, fixed with glutaraldehyde (2.5%), postfixed with aqueous osmium tetroxide (1%), and washed with PBS. The samples were dehydrated with ethanol, critical point dried, and coated with 3.5-nm gold palladium. A JEOL JSM-6330F SEM was used at 3 kV to acquire images.

For live cell immunostaining and imaging, live MDC1 were stained with 10 µg/ml Siglec-1-488 (CD169) Ab (Clone 7-239, Serotec) or isotype control-488 Ab (BD Biosciences) for 1h at 37 °C and 5% CO_2._ Non-specific mAb binding was blocked using media containing 25% FBS. MDC1 were then treated with rhCD40L for 10–12 h prior to staining for 2 h with 1 µM SiR-actin (Spirochrome AG, Stein am Rhein, Switzerland) at 37 °C and 5% CO_2_, and imaging. For Structured Illumination Microscopy (SIIM), live cell cultures were maintained in an imaging chamber at 37 °C and 5% CO2 in complete media (cIMDM) during image acquisition. Super-resolution images were collected in 3D SIM mode using live cells maintained at 37 °C in cIMDM using the AAA Nikon SIM and camera system at 100x magnification and 0.12 µm slices. Image reconstruction and analysis was carried out using NIS-ELEMENTS software.

### TNT inhibition

Mature MDC1 were harvested and plated at 2.5x10^5^ cells/ml in a final volume of 200µL/well of a flat-bottomed 96-well plate. Media alone or LatA (Invitrogen, Molecular Probes) was added to the cell suspensions at a final concentration of 100nM, the lowest concentration that effectively inhibited MDC1 reticulation and rhCD40L (0.5µg/mL) was added. After 24h of culture, images were collected, and individual image fields (≥ 10/condition) scored for the percentage of reticulation-positive cells, as described previously (23). To inhibit cholesterol synthesis, iMDC were treated with cytokine-containing maturation cocktail to generate MDC1 followed by the addition of 20 mM simvastatin (Sigma) in cIMDM for 2 days prior to harvest and subsequent exposure to rhCD40L for 24h.

### Soluble protein biomarker analysis

Soluble biomarker analysis was carried out using the MesoScaleDiscovery (MSD) multiplex platform. A custom U-plex 96-well plate was used to analyze soluble protein content in differentially activated MDC supernatants. To generate supernatants, MDC1, MDC1_CTL_, and MDC_PGE2_ cultures were established. On day 7 of culture, cells were harvested, plated on a 48-well plate (5x10^5^ cells/mL) and exposed for 48h to rhCD40L For the MDC1_CTL_ cocultures, CD8+ T cells were removed using a positive magnetic bead selection prior too plating them. After CD40L exposure, cells were pelleted by centrifugation to collect the supernatant, which was cryopreserved until multiplexing. Supernatants were prepared and analyzed according to manufacturer’s protocol. The MSD plate was read on the MESO QuickPlex SQ 120MM plate reader. For analysis, protein concentrations were normalized to 1x10^5^ cells/mL plated during CD40L incubation.

### Single-cell RNA sequencing

MDC1 and MDC_PGE2_ from an individual donor (final n = 3) were generated, washed to remove residual maturation cytokines, and stimulated with rhCD40L (1µg/mL) for 4 hours. The cells were then harvested and stained with condition-specific oligomer-conjugated multiplexing antibodies (BD Biosciences). Barcoded cells were then mixed in equal parts before loading onto a BD Rhapsody cartridge per manufacturer’s instructions for single cell capture using the BD Rhapsody express instrument. Quality control metrics were verified using the BD Rhapsody scanner. Prepared cDNA libraries of the whole transcriptome and barcoding antibodies, generated according to manufacturer protocol, were submitted to the Emory Yerkes National Primate Research Center Genomics Core for sequencing on an Illumina NovaSeq 6000. Initial cell calling, quality filtering, alignment, and annotation were performed using the BD Rhapsody Whole Transcriptome Analysis pipeline on the Seven Bridges Genomics platform. The resulting data were then imported into R Studio version 4.2.1, and Seurat version 5.0.0 was used for further quality control (32). After removing multiplets and cells with mitochondrial reads >25%, *n* = 52,492 cells were included in downstream analysis. Data were normalized via sctransform and integrated using the RPCA method to correct for batch effects before differential gene expression testing (33). The UpSetR package was used to visualize unique sets of differentially expressed genes (34). Pathway enrichment analysis was conducted using the pathfindR package version 2.3.1 (35). The EnhancedVolcano and SCpubr packages were used to generate volcano plots and uniform manifold approximation and projection (UMAP) plots respectively (36, 37).

### Statistics

**Figure 1:**
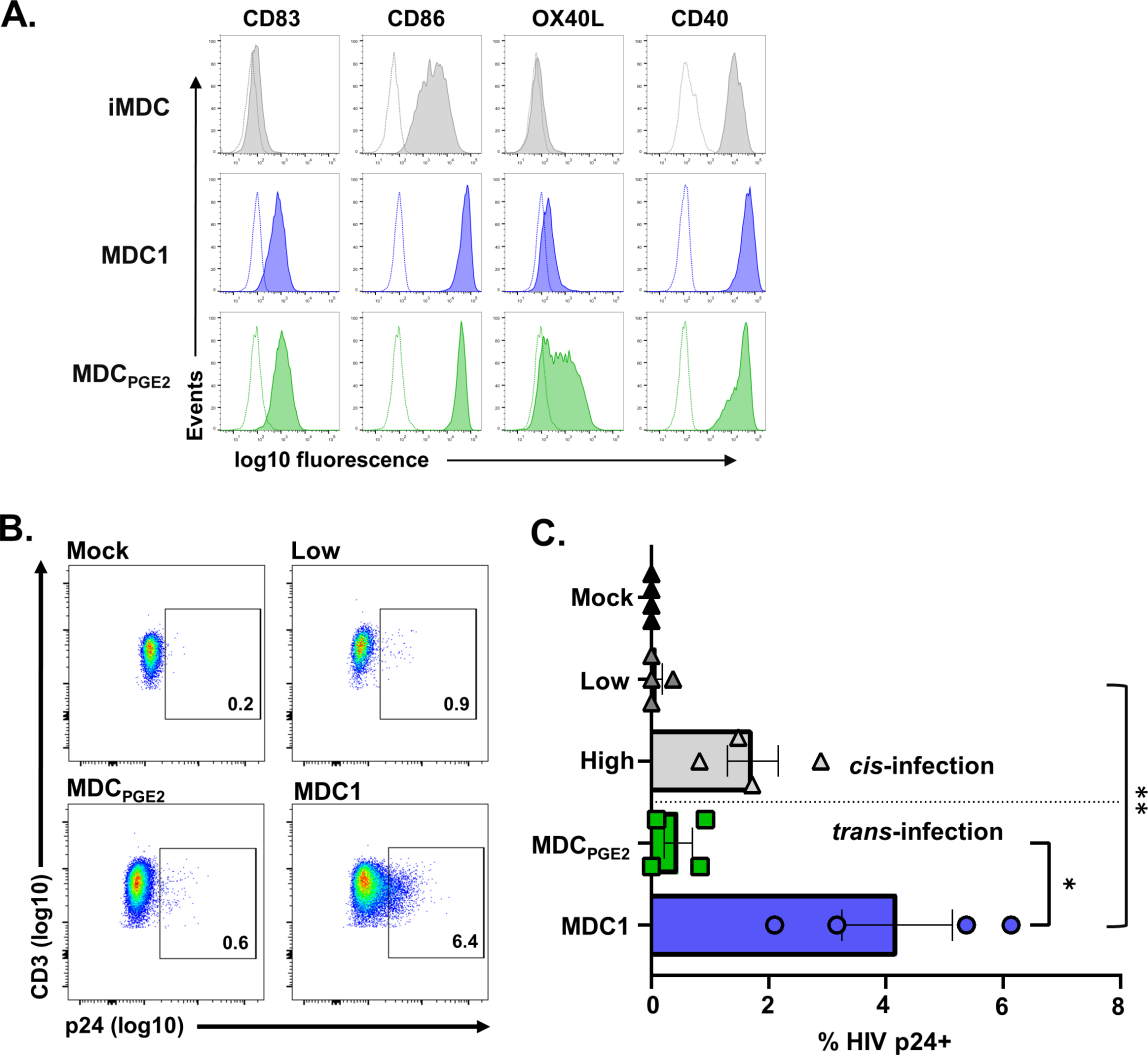
DC matured by mediators of type-1 immunity display enhanced capacity to facilitate HIV-1 transfer to CD4+ T cells. **(A)** Flow cytometry histograms of immature (iMDC) and differentially-matured (MDC1, MDC_PGE2_) monocyte-derived DC showing relative surface expression of CD83, CD86, OX40L, and CD40L. **(B)** Pseudocolor flow cytometry plots from a representative experiment depicting HIV-1 infections of autologous, preactivated CD4^+^ T cells including mock (top left), *cis*-infection (top right), MDC1 *trans*-infection (bottom left), and MDC_PGE2_ *trans*-infection (bottom right). Both MDC types were loaded with a low amount of input virus (10–^3^ MOI) prior to co-culture and CD4^+^ T cells were exposed to the same amount of input virus prior to culture in the *cis-*infection panel. **(C)** Combined data from independent *cis*- and *trans*-infection experiments using MDC1, MDC_PGE2,_ and autologous CD4^+^ T cells from different healthy donors. The y-axis labels “high” and “low” represent an MOI of 10–^1^ and 10^-3^, respectively. Data are represented as mean percentage of HIV-1 core Ag (p24) positive cells ± SE. *P*-value determined by Kruskal-Wallis test followed by Dunn’s multiple comparison test, n=4, *p < 0.05.

Statistical analyses were conducted using GraphPad Prism version 10.0.3. To determine normality for datasets, the Shapiro-Wilk test was conducted. Data following a normal distribution were analyzed using either the unpaired or paired Student’s t-test where stated. Data following a non-normal distribution (as in **Figure 1C**) were analyzed using the Kruskal-Wallis test. Where displayed, matched values were log transformed before statistical testing to reduce skew in datasets and analyze an approximately normal distribution. In all tests, P values of <0.05 were considered statistically significant. Data are represented as mean ± SEM. For experimental data generated by exposing MDC1 + CD40L + TNT blocking agents (LatA or Simv), the percentage of reticulation positive cells ± SE (defined as previously described (23)) is representative of 3 donors tested (>50 cells assessed/condition/donor sample), and statistical significance was determined using Fisher’s exact test. For the single cell analysis, differential gene expression was determined using MAST via Seurat::FindMarkers (38).

## Results

### Monocyte-derived DC matured by mediators of type-1 immunity display enhanced capacity to facilitate HIV-1 *trans*-infection of CD4^+^ T cells

We first established basic HIV-1 infection and readout methods by conducting a dose-response *cis*-infection of purified CD4^+^ T cells isolated from peripheral blood mononuclear cells (PBMC) collected from healthy, HIV-1 seronegative donors using an R5-tropic strain, HIV-1 BaL (**Supplementary Figure S2**). After 6 days, culture supernatants were collected to measure p24 core antigen by ELISA while cells were tested for intracellular p24 expression using flow cytometry. We found both detection methods revealed similar patterns of infection, e.g., higher input virus yielded greater p24 detection, and low virus input resulting in minimal infection. Given that flow cytometry enables detection of relative HIV-1 infection on a cellular level, we moved forward with this approach as our primary p24 detection method in subsequent experiments.

To first determine if enhanced HIV-1 *trans*-infection ability is a general trait of monocyte-derived DC matured under proinflammatory conditions, we generated functionally distinct, mature DC using previously established strategies (26, 28). Briefly, we employed a maturation cocktail of both type-I and type-II interferons, as well as a nucleic acid mimic, polyinosinic:polycytidylic acid (poly(I:C)), to yield proinflammatory type-1 polarized MDC (MDC1), defined by their surface expression of the maturation marker CD83, enhanced uniform expression CD86 and CD40 (as compared to iMDC), and low expression of OX-40L (**Figure 1A**, **Supplementary Figure S3A**) (27). In contrast, we added a classic maturation cocktail that includes prostaglandin E2 (PGE2), a mediator of chronic inflammation, to generate a subset of mature DC (MDC_PGE2_) also characterized by their surface expression of CD83, upregulated uniform expression of CD86 and CD40, as well as their increased expression of OX40L (**Figure 1A**, **Supplementary Figure S3A**) (27). Importantly, the MDC1 characteristically acquired a heightened capacity to produce IL-12p70 upon CD40L stimulation while the MDC_PGE2_ displayed a deficiency in their CD40L-induced IL-12p70 production (**Supplementary Figure S3B**.).

We then assessed the ability of these differentially matured DC to facilitate HIV-1 *trans*-infection to autologous CD4^+^ T cells in coculture after being exposed to a low concentration of virus (MOI 10^-3^) that we earlier determined to be ineffective at promoting *cis*-infection of pre-activated CD4^+^ T cells. In doing so, we found that the inflammatory MDC1 consistently displayed a higher capacity to mediate HIV-1 *trans*-infection to autologous CD4^+^ T cells than MDC_PGE2_ as determined by intracellular p24 expression using flow cytometry (**Figures 1C, D**). In fact, this mode of HIV-1 infection (MDC1 *trans*-infection) trended as being more effective than direct *cis*-infection the CD4+ T cells, even when the T cells were exposed to a 100 fold increase in HIV-1 concentration (MOI 10^-3^) (**Figure 1D**). To control for potential involvement of productive HIV-1 *cis*-infection of the DC, the HIV-1 exposed DC were also cultured with media alone, and supernatants were harvested to test for HIV-1 p24 by ELISA after 6 days.

### High expression of Siglec-1 enables enhanced HIV-1 binding and augmented *trans*-infection potential of type-1 proinflammatory MDC

**Figure 2:**
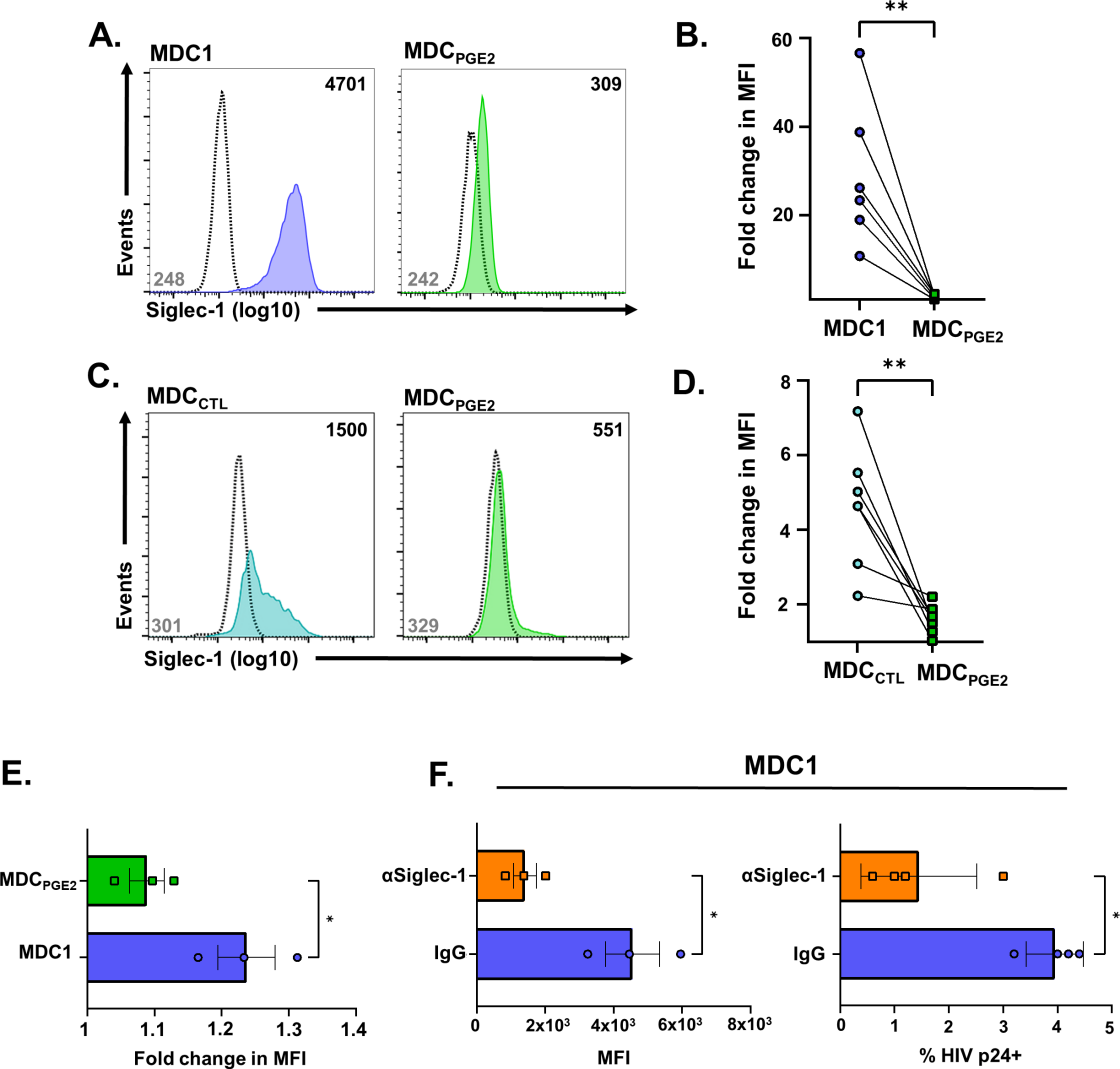
High expression of Siglec-1 enables enhanced HIV-1 binding and augmented *trans*-infection potential of type-1 proinflammatory MDC. **(A)** Histogram of surface expression of Siglec-1 on MDC1 and MDC_PGE2_, respectively, as characterized by flow cytometry. Colored peaks represent positive staining with Siglec-1-PE whereas grey peaks represent unstained FMO controls. Inset numbers indicate stained MFI (top right) or unstained MFI (bottom left). **(B)** Fold change of Siglec-1 MFI for MDC1 and MDC_PGE2_, respectively, compared to their unstained FMO controls. *P*-values were determined by paired Student’s t test, n=6, **p <.001. **(C)** Histogram of surface expression of Siglec-1 on MDC_CTL_ and MDC_PGE2_, respectively. **(D)** Fold change of Siglec-1 MFI for MDC_CTL_ and MDC_PGE2_, respectively, compared to their unstained controls. *P*-values were determined by paired Student’s t test, n=7, **p<.01. **(E)** MDC1 and MDC_PGE2_ were incubated with a replication-deficient, GFP-expressing HIV-1 strain (HIV-GFP) at an MOI of 10–^3^ to assess differences in binding by flow. Data are represented as percent HIV-GFP+ ± SE. *P*-values were determined by unpaired Student’s t test, n=3, *p<0.05. **(F)** Siglec-1 on MDC1 was blocked using an anti-Siglec-1 antibody (1µg/mL) or MDC1 were treated with matched isotype control (1µg/mL) to determine the effect on HIV-GFP binding (left panel, n=3) or HIV-1 BaL *trans*-infection (right panel, n=4). Data are represented as percent HIV-GFP+ or HIV-1 core Ag (p24) positive cells ± SE. *P*-values were determined by paired Student’s t-test, *p < 0.05.

Previous studies indicate that MDC capture HIV-1 primarily using the I-type lectin Siglec-1, which is significantly upregulated upon lipopolysaccharide or type-I IFN activated DC (15, 16, 18, 39). To understand if the known role of Siglec-1 in *trans*-infection extends to type-1 MDC, we investigated differentially matured DC for their expression of Siglec-1 using flow cytometry. When comparing MDC1 to MDC_PGE2_, the surface expression of Siglec-1 was significantly higher on MDC1, a pattern which was consistent among six different donors tested (**Figures 2A, B**). We next wanted to determine if a similar phenotypic pattern would appear in high IL-12p70 producing MDC generated through the helper activity of CTL (MDC_CTL_) using a previously described method from our group (24, 29). By co-culturing iMDC with autologous CD8^+^ T cells in the presence of OKT3, an anti-CD3 antibody that binds to iMDC via Fc-rec and promotes TCR engagement and cellular immune cross-talk, we confirmed the generation of this MDC_CTL_ phenotype (**Supplementary Figures S3A, B**), and determined that MDC_CTL_, like MDC1, also express significantly higher Siglec-1 than their standard, PGE2 matured counterpart (**Figures 2C, D**).

To confirm that the enhanced Siglec-1 expression on MDC1 results in efficient virus capture, we measured binding of virus using a replication deficient, GFP-expressing HIV-1 strain (HIV-GFP). Flow cytometry revealed that MDC1 consistently capture more HIV-1 compared to MDC_PGE2_ (**Figure 2E**). Moreover, exposing MDC1 to an anti-Siglec-1 blocking antibody significantly reduced the ability of MDC1 to bind HIV (**Figure 2F**, left panel). Finally, we assessed whether blocking of Siglec-1 would influence MDC1-mediated *trans*-infection using the same Siglec-1 inhibitor. MDC1 were exposed to either anti-Siglec-1 or IgG isotype control antibodies, loaded with HIV-1, then placed into coculture with CD4^+^ T cells and tested in our *trans*-infection model. In these assays, we observed that the Siglec-1 blockade significantly inhibited the ability of MDC1 to mediate HIV-1 *trans*-infection of autologous CD4^+^ T cells (**Figure 2F**, right panel). These results are consistent with reports recognizing the mechanistic role of Siglec-1 in DC-mediated HIV-1 *trans*-infection (15, 18). Overall, our data support the notion that inflammatory signals induce a type-1 MDC phenotype with heightened Siglec-1 expression that contributes to a superior capacity to capture and disseminate HIV-1.

### The T helper signal CD40L amplifies HIV-1 *trans*-infection activity of MDC1

**Figure 3:**
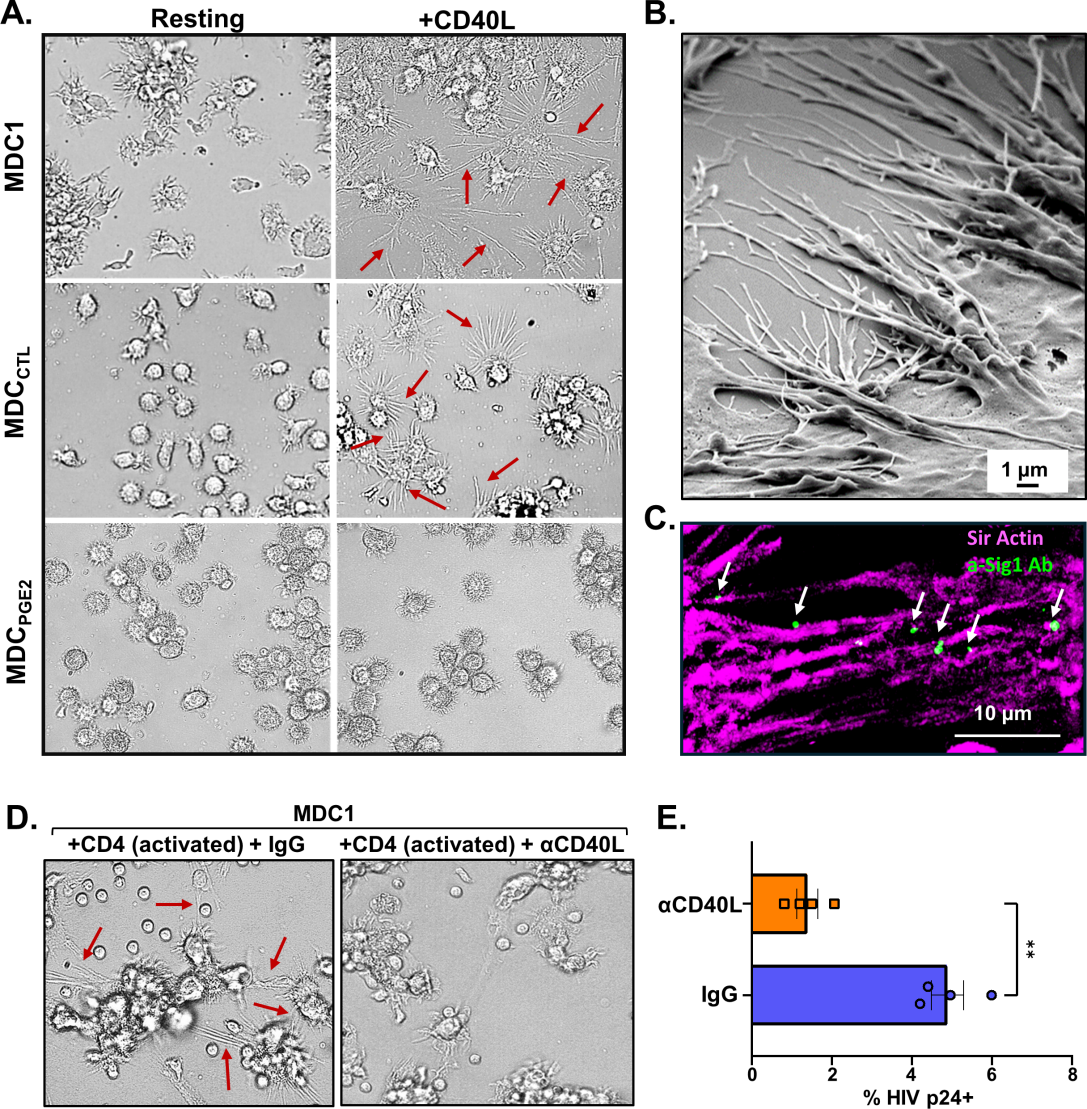
T helper signal CD40L amplifies HIV-1 *trans*-infection ability of MDC1. **(A)** Bright field images (40X) of MDC1 (top), MDC_CTL_ (middle), and MDC_PGE2_ (bottom) in the presence (right) or absence (left) of rhCD40L. Images have been sharpened to allow for visualization of TNT induced by CD40L. Red arrows highlight TNTs. **(B)** Scanning electron micrograph of MDC_CTL_ showing induction of TNT following 18h exposure to rhCD40L. **(C)** SIM-acquired image showing Siglec-1 expression (green) along CD40L-induced TNTs on MDC1 that are illuminated using the F-actin probe, SiR Actin (magenta). **(D)** Brightfield images (40X) of MDC1 co-cultured with activated CD4^+^ T cells in the absence (left) or presence (right) of anti-CD40L blocking antibody. Images have been sharpened to enable visualization of TNTs induced in a MDC1/CD4 coculture, indicated by red arrows. **(E)** MDC1-mediated HIV-GFP *trans*-infection in the presence of anti-CD40L inhibitor or IgG isotype control. *P*-value was determined by paired Student’s t test, n=4, **p < 0.01.

Major differences between MDC1 and MDC_PGE2_ lie in the character of their response to the Th signal CD40L. For example, in addition to heightened IL-12p70 production, CD40L uniquely and dramatically alters the morphology of MDC1. Our group has previously shown that CD40L stimulation results in a process of reticulation wherein MDC1, unlike MDC_PGE2_, rapidly form interconnected networks of TNTs that promote their surface area, reach, and their ability to exchange intracellular content (23). This CD40L-induced reticulation process in MDC1 (**Figure 3A**, top row) and lack thereof in MDC_PGE2_ (**Figure 3A**, bottom row) is evident using standard light microscopy. Similar to MDC1, CD40L also induced TNT networks in MDC_CTL_ (**Figure 3A**, middle row), the complexity of which is revealed in striking detail in scanning electron microscopy (SEM) images depicting ultrafine branched TNTs (**Figure 3B**). Moreover, using live cell enhanced live-cell structured illumination microscopy SIM, we demonstrated Siglec-1 antibody clustering and trafficking along CD40L-induced TNTs in MDC1 (**Figure 3C**). Importantly, we also observed evidence of reticulation and TNT formation in our MDC1/CD4^+^ T cell coculture model (**Figure 3C**, left panel), which is inhibited with the addition of a CD40L blocking antibody (**Figure 3C**, right panel). We also note as expected that the characteristic IL-12p70 production is heightened in our coculture model, and that blocking with the same anti-CD40L antibody diminishes this cytokine production (**Supplementary Figure S3C**).

Given that there is little information about the role of CD40L in HIV-1 *trans*-infection despite it being a critical Th cell feedback signaling factor with major influence on MDC functionality, we hypothesized that the CD40L/CD40 interaction between CD4^+^ T cells and type-1 proinflammatory MDC1 contributes mechanistically to the *trans*-infection process. To test this, we incorporated the anti-CD40L blocking antibody in our *trans*-infection coculture, which resulted in a significant attenuation in the *trans*-infection activity of the MDC1 (**Figure 3D**). These data collectively indicate that CD40L signaling events occurring during immunologic crosstalk between type-1 proinflammatory MDC and CD4^+^ T cells play a mechanistic role in facilitating HIV-1 *trans*-infection.

### CD40L-driven MDC1 reticulation supports HIV-1 *trans*-infection

**Figure 4:**
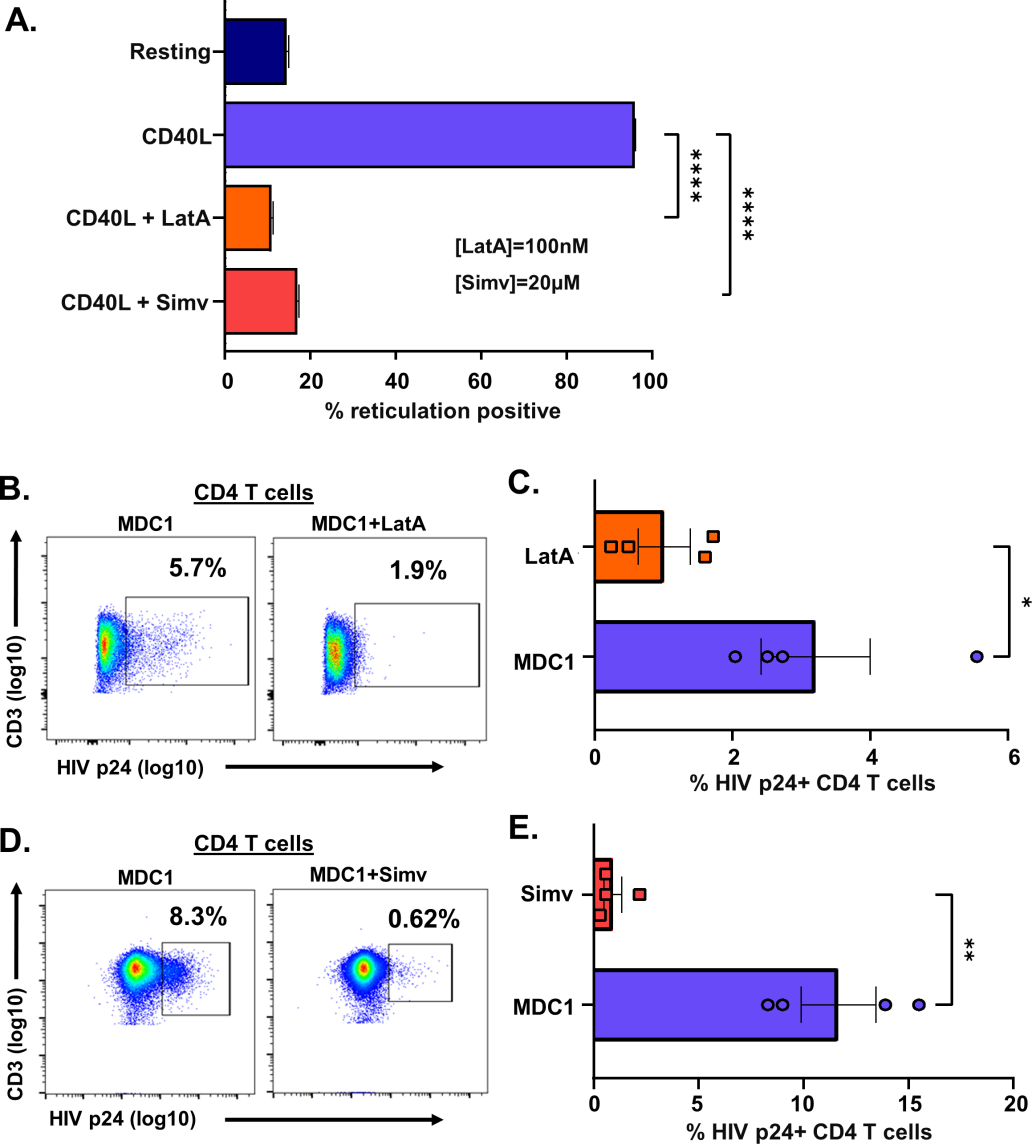
CD40L-driven MDC1 reticulation supports HIV-1 *trans*-infection. MDC1 were treated with either the F-actin depolymerization drug latrunculin A (LatA; 100nM) or the cholesterol inhibitor simvastatin (Simv; 20µM) and assessed for their ability to form TNT networks or facilitate HIV-1 *trans*-infection. **(A)** Ten random image fields were collected and used to quantify CD40L-induced DC reticulation as previously described (23). Data represents the percentage of reticulation positive cells ± SE from one representative of three donors tested (>75 cells assessed per condition). *P*-values were determined by Fisher’s exact test, ****p < 0.0001. **(B)** Pseudocolor flow cytometry plots from a representative experiment depicting MDC1-mediated HIV-1 *trans-*infection of autologous CD4+ T cells and the inhibitory impact of LatA (right), with **(C)** representing a summary of 4 independent experiments described in **(B) (D)** Flow cytometry plots from a representative experiment depicting the inhibitory impact of Simv (right) on the ability of MDC1 to mediate HIV-1 *trans*-infection, with **(E)** representing a summary of 4 independent experiments described in **(D)** *P*-values were determined by paired Student’s t test, n=4, *p<0.05 and **p<0.01.

We next set out to determine whether the CD40L-induced TNT formations that occur as an exclusive trait of type-1 polarized MDC play a role in our HIV-1 *trans*-infection model. Several reports highlight the role of actin stability and actin-rich membrane extensions in HIV-1 *trans*-infection by DC as well as macrophages, a related myeloid cell type (40–43). Likewise, we sought approaches to inhibit TNT formations in our inducible MDC1 reticulation and *trans-*infection models and chose two methods to evaluate, including the use of latrunculin A (LatA) and the cholesterol inhibitor simvastatin (simv). LatA effectively prevents F-actin assembly, an important component of TNT, without significantly affecting cell viability (44). Simvastatin was included in our studies because of its ability to inhibit early stages of cholesterol biosynthesis, and cholesterol-rich membrane nanodomains are required for TNT stability as they have been detected along the surface of TNT and result in destabilization if depleted (45). When MDC1 were treated with either LatA or simvastatin, the CD40L-induced reticulation events were significantly inhibited (**Figure 4A**). Next, we determined whether blocking TNT with LatA or simvastatin treatments before HIV-1 exposure and coculture with CD4^+^ T cells would alter MDC1-mediated *trans*-infection in separate experiments. Indeed, when TNT formation was blocked with either inhibitor, a significant reduction in MDC1 *trans*-infection activity was consistently observed (**Figures 4B, C**). Taken together, these results support our hypothesis that CD40L contributes to type-1 MDC-mediated *trans*-infection wherein CD40L-induced TNT represent an important pathway for virus dissemination from MDC1 to CD4^+^ T cells.

### CD40L-induced CCL20 enhances susceptibility of CD4^+^ T cells to HIV-1 infection

**Figure 5:**
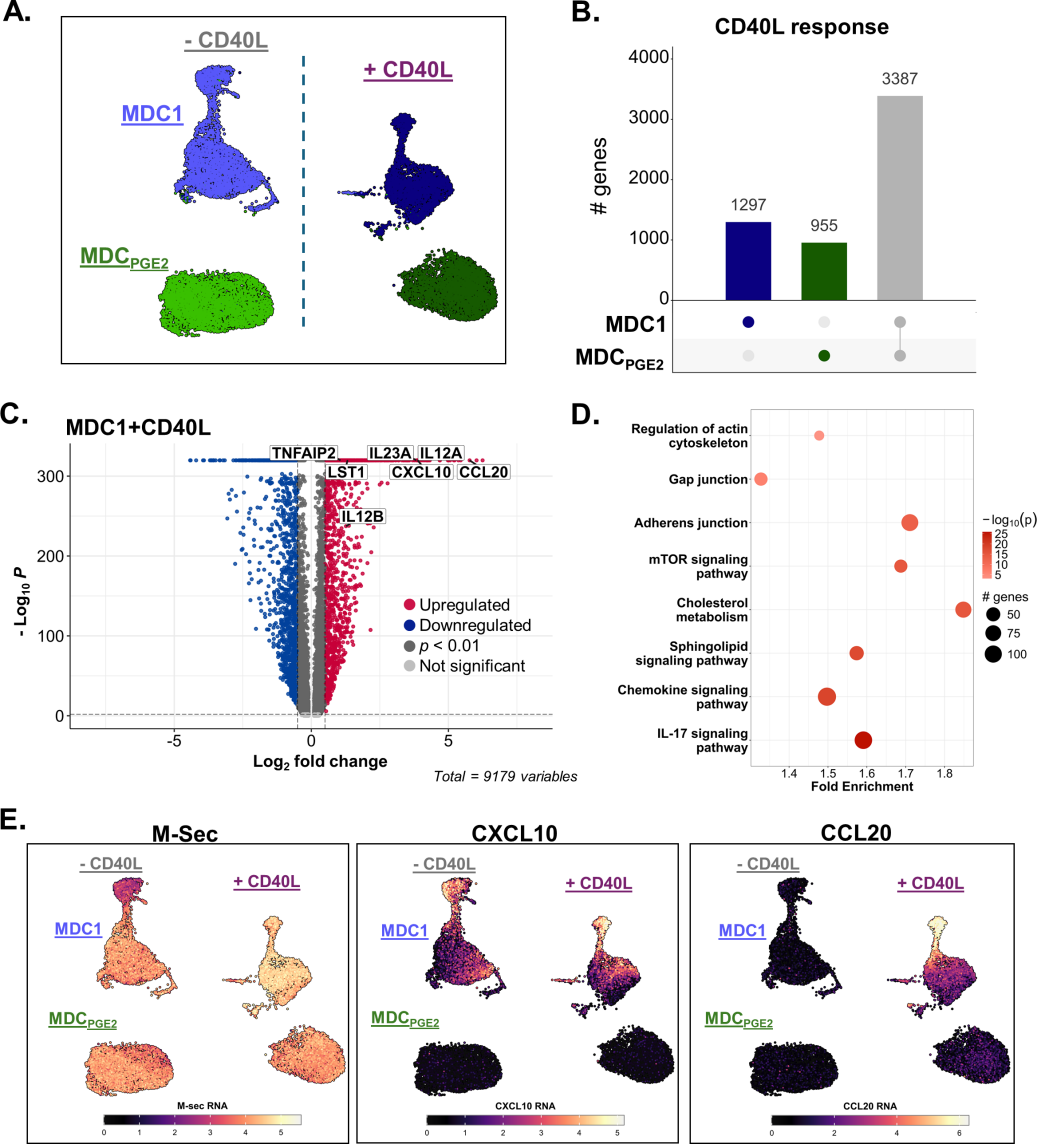
Single cell transcriptomic analysis reveals unique responsiveness of MDC1 to CD40L. Single cell RNA sequencing was used to compare the whole transcriptomes of the MDC types, both with and without 4hr of rhCD40L stimulation (n=3 donors). **(A)** UMAP plot of a representative donor color-coded by condition. **(B)** Genes differentially expressed by MDC1 in response to CD40L treatment were compared to those differentially expressed by MDC_PGE2_ and visualized in an UpSet plot. The unique gene set expressed by MDC1 responding to CD40L appears in blue, while the MDC_PGE2_ set appears in green. **C)** Volcano plot identifying genes up- and downregulated in the MDC1 + CD40L condition compared to all other conditions. Adjusted *P*-values were determined using MAST. **(D)** KEGG pathways enriched in MDC1 + CD40L as determined by pathfindR analysis. **(E)** Expression of the TNT-associated gene M-Sec (*TNFAIP2*) and the chemokines *CXCL10* and *CCL20* overlaid on the UMAP plot of the same representative donor depicted in **(A)**.

While the character of response to the Th cell factor CD40L varies between differentially matured DC subsets, CD40L has a broad range of effects on DC function and is nevertheless a potent signal for all the DC types tested in our study. Because of the multifaceted impact of CD40L on DC function, we were interested in exploring additional MDC1 intrinsic responses to CD40L that might uniquely contribute to their enhanced *trans*-infection capacity. We thus performed whole transcriptome single-cell RNA sequencing to identify transcriptional differences between MDC1 and MDC_PGE2_ as well as changes occurring in these subsets following stimulation with CD40L. Uniform manifold approximation projection (UMAP) visualization identified 4 distinct clusters corresponding to the treatment conditions (**Figure 5A**). Comparison of genes differentially expressed by MDC1 and MDC_PGE2_ when treated with CD40L revealed gene sets unique to each, further underscoring that both subsets do respond to this signal (**Figure 5B**). When compared to all other conditions, MDC1 treated with CD40L differentially transcribed 9,179 genes. This gene set included increased expression of *TNFAIP2* (also known as M-Sec) and *LST1* (**Figure 5C**), both of which are known to promote TNT formation (46). Pathway enrichment analysis of the MDC1+CD40L markers also identified enriched terms associated with regulation of actin cytoskeleton, gap junction formation, and cholesterol metabolism (**Figure 5D**). These pathways each have components strongly linked to TNTs, including gap junctions, where TNTs have been demonstrated to augment HIV-1 spread via functional gap junctions established between distant cells (47).

**Figure 6:**
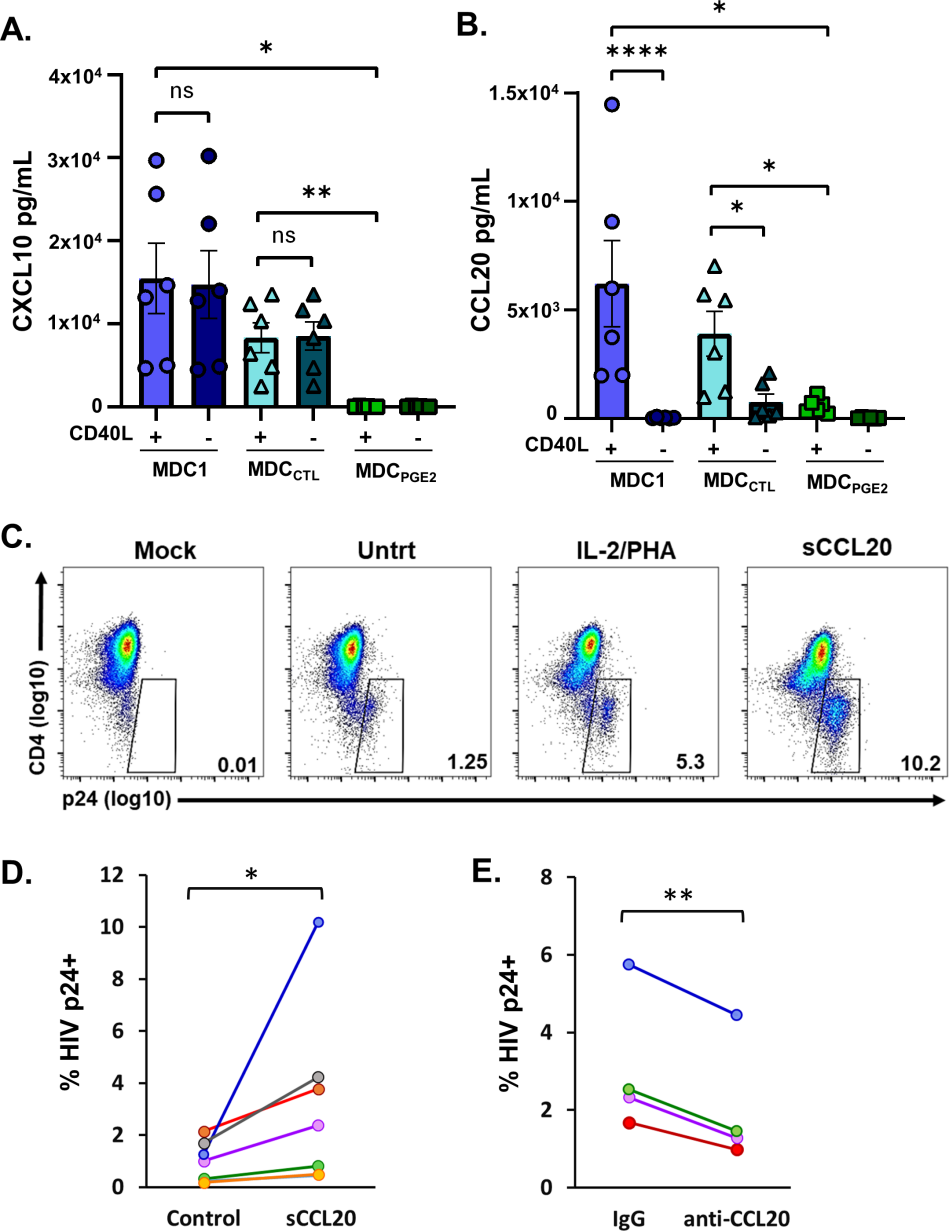
CD40L-induced CCL20 enhances susceptibility of CD4^+^ T cells to HIV-1 infection. **(A)** Mature DC were tested for their production of CXCL10 in response to 48hr of rhCD40L stimulation. Error bars represent ± SE. *P*-values were determined by one way ANOVA followed by Holm-Šídák’s multiple comparisons test, ns, ***p < 0.001. **(B)** Mature DC were tested for their production of CCL20 in response to 48hr of rhCD40L stimulation. Error bars represent ± SE. *P*-values were determined by one way ANOVA followed by Holm-Šídák’s multiple comparisons test, n=6, *p < 0.05, **p<0.01, ***p<0.001, and ****p <.0001. **(C)** Pseudocolor flow cytometry plots from a representative experiment of 4 performed HIV-1 *cis-*infections (10–^1^ MOI) of 48h pre-treated CD4^+^ T cells including (from left to right) mock, untreated, IL-2/PHA treated, and soluble CCL20 treated CD4^+^ T cells after day 6 of culture. **(D)** Combined data from 6 independent experiments assessing the effect of soluble CCL20 on *cis*-infection. Data are represented as mean percentage of HIV-1 core Ag (p24) positive cells with lines connecting matched donor paired values. Student’s paired t-test, n=6, *p < 0.05. **(E)** Combined data from 4 independent experiments assessing the effect of MDC1-derived CCL20 on *cis*-infection. CD40L-activated MDC1 culture supernatant containing induced CCL20 was treated with an anti-CCL20 blocking antibody or an IgG control and subsequently to exposed to CD4^+^ T cell cultures 48hr prior to HIV-1 infection. *P*-values determined by paired Student’s paired t-test, n=4, **p<0.01.

Besides TNT-associated terms, CD40L-activated MDC1 also demonstrated a 1.5-fold increase in chemokine signaling (**Figure 5D**). The genes upregulated in this pathway included a 3.88 log_2_ fold increase of *CXCL10* and a 5.77 log_2_ fold increase of *CCL20* (**Figure 5C**). The unique upregulation of these chemokine transcripts in MDC1 upon exposure to CD40L was of particular interest because CXCL10 and CCL20 have each previously been linked to the establishment of latent HIV-1 infection in resting CD4^+^ T cells *in vitro* (48). UMAP clustering revealed a particular subset of MDC1 strongly upregulating these chemokines in response to CD40L, and the effect was most pronounced for *CCL20* (**Figure 5E**). Importantly, MDC_PGE2_ do not display this response. To confirm our sequencing data at the protein level, supernatants from MDC1, MDC_CTL_, and MDC_PGE2_ cultures were analyzed using MesoScaleDiscovery (MSD) multiplexing. MDC1 and MDC_CTL_ both produced significantly higher levels of CXCL10 than MDC_PGE2_; however, CXCL10 release was unaffected by CD40L stimulation (**Figure 6A**). Conversely, CCL20 protein production was in congruency with the RNA-level data where protein release was highest in both type-1 MDC subsets, particularly upon secondary exposure to CD40L (**Figure 6B**).

Given its high level of induction, we chose to further explore how CD40L-induced release of CCL20 by type-1 MDC might influence HIV-1 infection of CD4^+^ T cells in our model. As mentioned earlier, CCL20 has been previously implicated in the successful establishment of latent HIV-1 infection in resting CD4^+^ T cells (48). We were therefore interested in testing the effect of CCL20 on the susceptibility of CD4^+^ T cells to HIV-1 *cis*-infection. Purified, resting CD4^+^ T cells were exposed to soluble CCL20 for 48h before exposure to HIV-1 BaL. We observed that infection was heightened in cells exposed to CCL20, indicating this factor enhances CD4^+^ T cell susceptibility to infection (**Figures 6C, D**). To recapitulate these results with MDC1-derived CCL20, supernatant from a CD40L-activated MDC1 culture that was determined to contained CCL20 (**Figure 6B**) was pre-treated with an anti-CCL20 blocking antibody or an IgG control. CD4^+^ T cells were then treated with these supernatants prior to their exposure to HIV-1 in our *cis*-infection model. Interestingly, we discovered that blocking MDC1-released CCL20 in supernatant prior to exposure to CD4^+^ T cells resulted in an approximately forty percent reduction in HIV-1 infection (**Figure 6E**). These data collectively indicate that in addition to efficiently mediating the intercellular transfer of HIV-1, proinflammatory type-1 MDC have the potential to enhance bystander CD4^+^ T cell susceptibility to HIV-1 targeted infection through their unique instructive release of CCL20 in response to the Th cell feedback signal CD40L.

## Discussion

It is well known that both immature and mature DC subsets can facilitate HIV-1 *trans*-infection of CD4^+^ T cells through different mechanisms (7, 9–11, 15). However, most of the reports to date have limited the mechanistic focus of DC-mediated *trans*-infection to the general maturation status of the DC (immature vs mature) or single mechanistic aspects such as those related to a host cell receptor and HIV-1 binding affinity. The role of environmental instruction in inducing phenotypically and functionally distinct DC subsets has been thoroughly described, with the combination of immune input signals derived directly from pathogens or indirectly from neighboring cells all contributing to the immune programming of DC and the character of the adaptive immune responses they subsequently induce (5, 26, 28). Given their central role in immunity, it is plausible that pathogens would evolve to target the function of DC for their own advantage. We and others have previously described how subtle changes to evolving HIV-1 epitope variants can result in a skewed inflammatory cytokine recall response by CTL that lack the ability to effectively eliminate the infected targets through cytolytic means (24, 49, 50). In addition to evading direct CTL killing, this partial immune escape and persistent CTL activity contributes to the environmental programming of pro-inflammatory MDC (MDC_CTL_) that acquire an enhanced capacity to drive HIV-1 *trans*-infection. In this scenario, the virus utilizes both the partial agonist activity of the CTL and the innate responsiveness of DC to environmental cues to drive HIV-1 dissemination and persistence.

In this study using specific strategies to generate differentially polarized MDC subtypes, we further demonstrate that the nature of the environmental activation signals that drive DC maturation greatly influences the ability of MDC to mediate HIV-1 *trans*-infection. We show that exposure of iMDC to a combination of pro-inflammatory signals translates into the generation of high IL-12p70 producing MDC1 with enhanced HIV-1 *trans*-infection capacity. However, when we use other activation factors to mature DC such as PGE2, a mediator of chronic inflammation that is known inhibit IL-12p70-driven type-1 immunity, this leads to the generation of MDC that are phenotypically mature yet considerably less effective at mediating HIV-1 *trans*-infection. These findings parallel our previous report showing the efficient HIV-1 *trans*-infection by pro-inflammatory MDC arising from the selective CTL helper activity induced by partial agonist HIV epitope variants mentioned above. Moreover, by coculturing iMDC with autologous CD8^+^ T cells in the presence of anti-CD3 monoclonal antibody (OKT3), we were able to recapitulate this CTL helper activity to drive the maturation and differentiation of MDC having the proinflammatory MDC_CTL_ functional phenotype that we previously described (51) to continue studies exploring the mechanistic aspects behind the enhanced *trans*-infection capacity of MDC_CTL_ and MDC1.

Whether matured using a cocktail of soluble inflammatory factors or through the helper activity of cytokine-producing CTL, the enhanced surface expression of Siglec-1 was a common characteristic among the MDC1 and MDC_CTL_, both of which have significant HIV-1 *trans*-infection potential. We confirmed through blocking studies that high cell surface expression of Siglec-1 confers the augmented ability to capture HIV-1 and mediate *trans*-infection of CD4^+^ Th cells. These findings are concurrent with earlier reports attributing Siglec-1 on LPS-matured DC to their ability to *trans*-infect (15, 16, 43, 52). Notably, the expression of Siglec-1 was significantly diminished in MDC that were generated in the presence of PGE2, a mature subset, which also proved to be poor at facilitating HIV-1 *trans*-infection. It is worth noting that the MDC_PGE2_ did demonstrate some degree of HIV-1 binding (although less than half of MDC1), presumably through their moderate surface expression of other molecules associated with HIV binding including ICAM-1 and DC-SIGN, which we show in **Supplementary Figure S3C**. Yet, the MDC_PGE2_ are notably far less efficient at *trans*-infection, suggesting that mechanistic involvement Siglec-1 binding in HIV uptake and transfer is particularly unique and important in the *trans*-infection process. Importantly, our results highlight that the nature of environmental cues received by DC during maturation is more critical to their capacity to drive HIV-1 *trans*-infection than merely their general maturation status.

Additionally, a critical and novel aspect of our current findings lies in the active role that CD4^+^ T cells have in the *trans*-infection process. Upon encounter with the MDC, the CD4^+^ T cell provides an important feedback signal to the MDC in the form of CD40L. While it is known that CD40/CD40L interaction serves many important immunologic functions, our study demonstrates that the CD40L signal can contribute to the process of viral transfer from MDC1 to CD4^+^ T cells, and that blocking the CD40/CD40L interaction in our assays reduces MDC1-mediated *trans*-infection. Again, the character of the responsiveness of the MDC to the CD40L signal plays a major role in the outcome of these viral transfer events, which is ultimately dictated by the innate immune imprinting during maturation. While all DC types tested were clearly responsive to the CD40L activation signal based on scRNA-seq analysis, the type-1 polarized monocyte-derived DC (MDC1 and MDC_CTL_) respond uniquely to this Th cell-derived signal by undergoing a process of reticulation, where they form extensive networks of TNT formations. We previously showed that this Th cell-driven immunologic mechanism increases the surface area and reach of the DC allowing for enhanced intercellular communication as well as transfer of antigen between interacting DC (23). Moreover, we show that Siglec-1 clusters along these CD40L-induced F-actin-rich TNT structures, like those which have been readily implicated in the intercellular transfer of pathogens as well as HIV-1 *trans*-infection of CD4^+^ T cells (42, 53–55). In this study, we show that impeding this dramatic immunologic cellular process of DC reticulation by blocking F-actin polymerization or cholesterol biosynthesis using LatA or simvastatin, respectively, results in considerable reduction of their potential to mediate HIV-1 *trans*-infection of CD4^+^ T cells.

In addition to driving TNT formation, our multiomic analysis further revealed that CD40L induces the production and release of the chemokine CCL20 as a distinctive trait among the type-1 programmed MDC1 and MDC_CTL_. Of importance, we showed that treatment of CD4^+^ T cells with CCL20 resulted in their increased baseline susceptibility to productive HIV-1 infection upon subsequent exposure to cell free virus. This effect of CCL20 is in accord with prior reports describing the role of CCL20 in the establishment and persistence of the latent viral reservoir in resting CD4^+^ T cells (48, 56). While CCL20 is a known ligand for the CCR6 chemokine receptor commonly associated with both Th1 and Th17 cells (57), it is unclear if HIV-1 is selectively infecting any functional CD4^+^ T cell subset in our *trans*-infection model. A confounding factor in our model is that we show CCR6 being downregulated upon exposure to CCL20 (**Supplementary Figures S3E, F**), making such determinations and interpretations difficult. Interestingly, the chemotactic interactions of CCL20/CCR6 have been shown to be interrupted by statins including simvastatin (58). Therefore, the inhibitory impact of simvastatin that was demonstrated in our *trans*-infection model might involve its negative effect on CCL20 responsiveness in addition to TNT formation. Further investigation is necessary to better understand the mechanisms of how the MDC-derived CCL20 signal increases T cell permissiveness to infection, the role of CCR6 expression on CD4^+^ T cells being targeted, and details of how this process may contribute to the establishment of the latent HIV-1 reservoir in different CD4^+^ T cell subsets during natural infection.

An important limitation to our study is that it focusses only on DC derived from blood-derived monocyte precursors, which differ functionally and transcriptionally from other primary tissue-derived conventional DC subtypes, and may or may not accurately represent fully differentiated lymph node homing blood/monocyte derived DC previously described by Ralph Steinman’s group as being a major responding DC type *in vivo* during acute infection (59). It is therefore unclear to what degree our findings would apply to these DC subsets and therefore warrants further ex-vivo and/or *in-vivo* investigative studies.

Taken together, our *in vitro* study advances the understanding of how environmental immune instruction supports or attenuates MDC-mediated HIV-1 *trans*-infection while uncovering the mechanisms by which HIV-1 exploits both DC function and the adaptive immune response to enhance viral spread. Ultimately, our results implicate the Th helper cell factor CD40L in DC-mediated *trans*-infection in part by its induction of TNTs. They further demonstrate that via induction of chemokine release, DC can also impact HIV-1 infection beyond facilitating the physical transfer of virus by attracting and priming CD4^+^ T cells for enhanced susceptibility and receptiveness to HIV infection.

## Data Availability

Supporting data from this manuscript will be made readily available by the authors. All single cell RNA sequencing data generated in this study are deposited in the dbGaP controlled access database under the accession number phs004749.v1 in accordance with IRB requirements. All code used for analysis will be made available for those interested.
