# Supplementary figures and images for: Type-1 inflammatory imprinting and programmed responsiveness to CD40L enhance Siglec-1-dependent HIV-1 trans-infection by dendritic cells
Source: Front Immunol. 2026 Jun 4;17:1834769. doi: 10.3389/fimmu.2026.1834769 (PMC13275350; doi:10.3389/fimmu.2026.1834769)

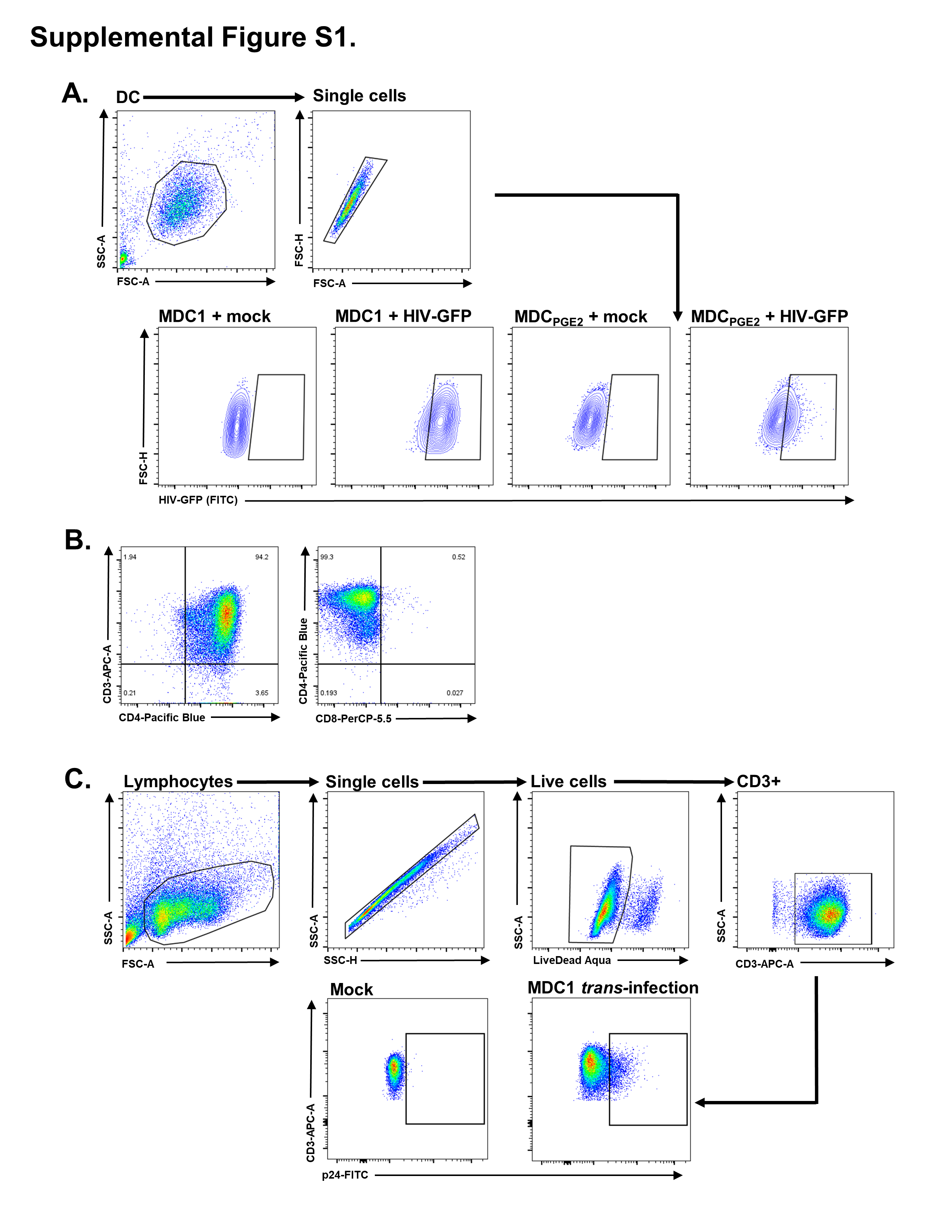

Supplement: Supplementary file 1 [file Image1.tif]

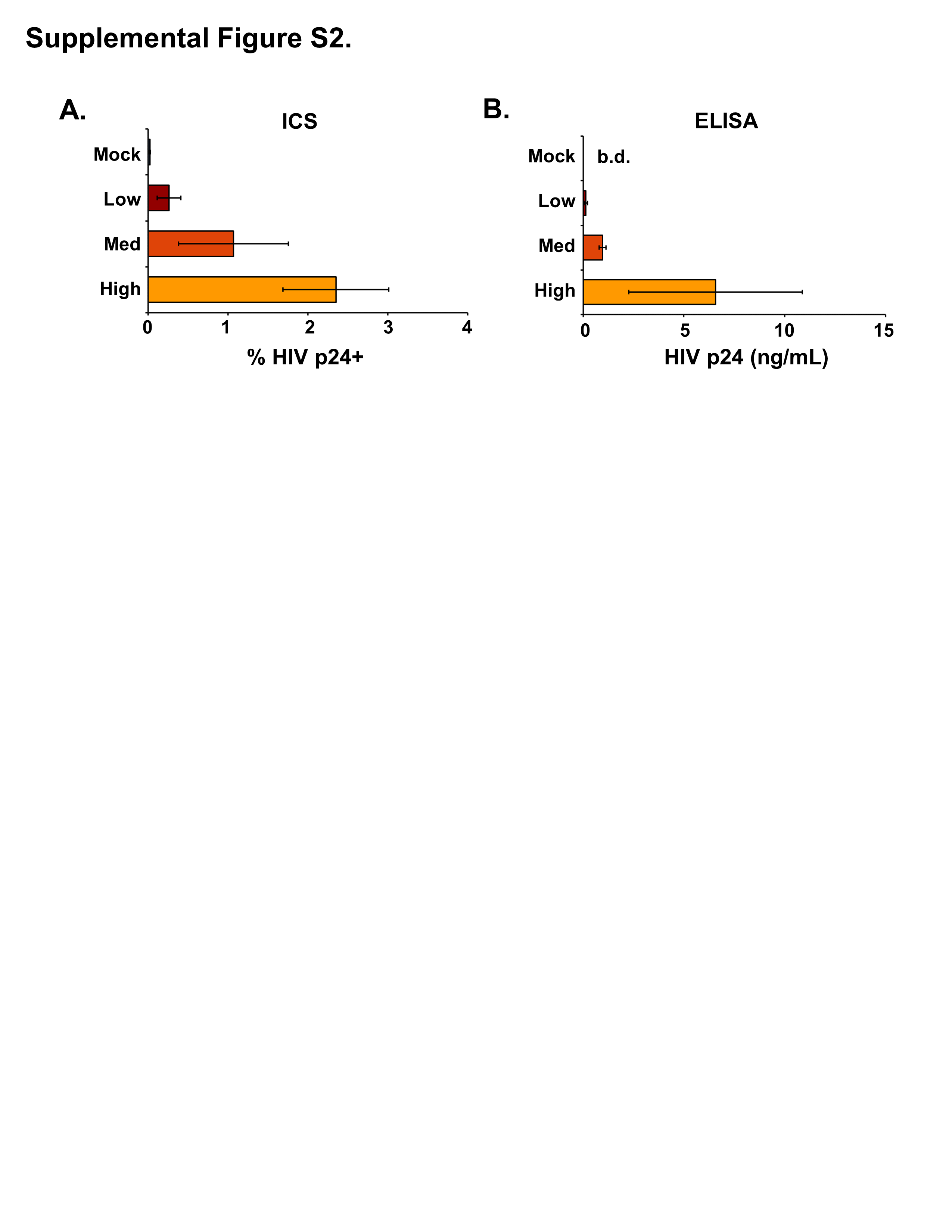

Supplement: Supplementary file 2 [file Image2.tif]

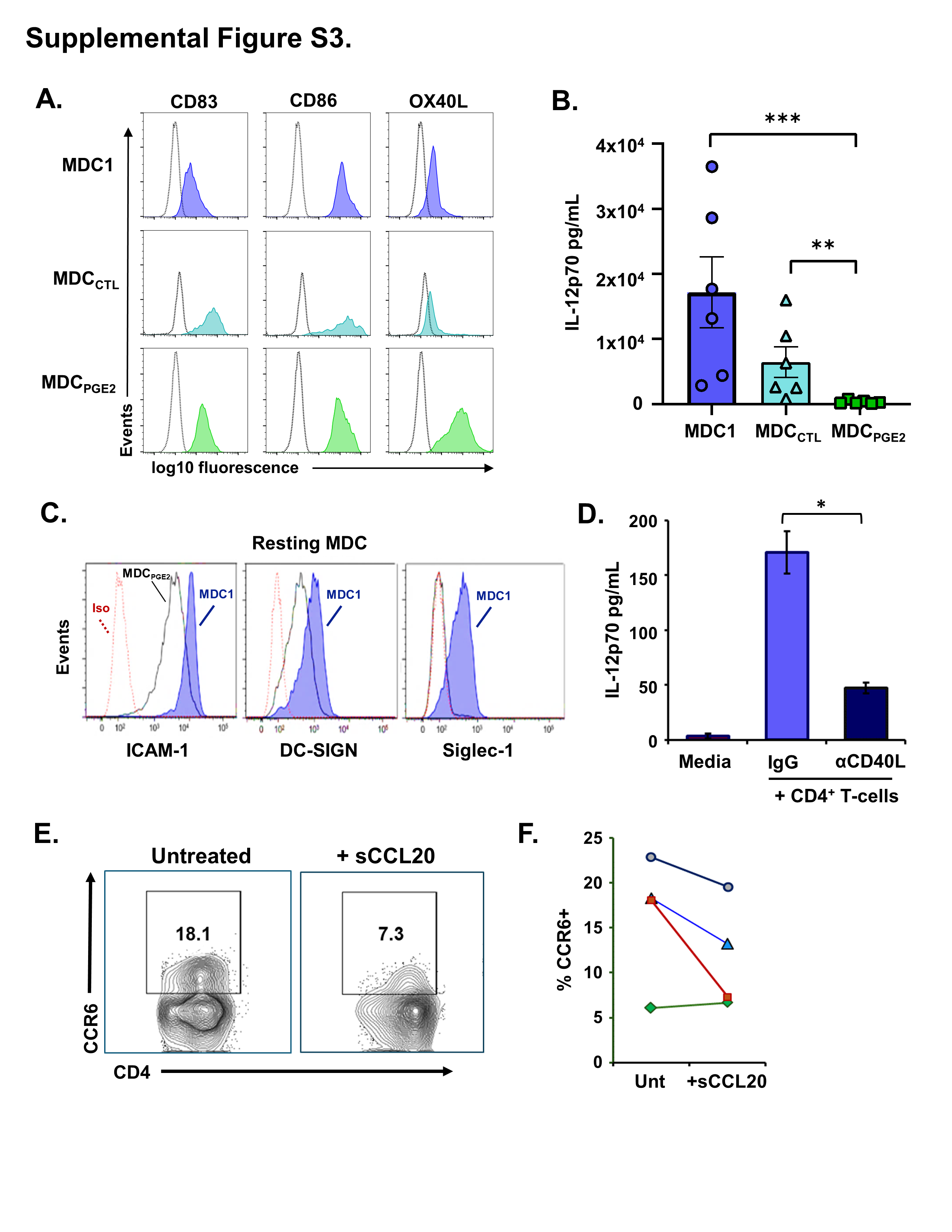

Supplement: Supplementary file 3 [file Image3.tif]
